# Provider Continuity Prior to the Diagnosis of Advanced Lung Cancer and End-of-Life Care

**DOI:** 10.1371/journal.pone.0074690

**Published:** 2013-09-03

**Authors:** Gulshan Sharma, Yue Wang, James E. Graham, Yong-Fang Kuo, James S. Goodwin

**Affiliations:** 1 Division of Pulmonary and Critical Care Medicine, Department of Internal Medicine, University of Texas Medical Branch, Galveston, Texas, United States of America; 2 Department of Internal Medicine, University of Texas Medical Branch, Galveston, Texas, United States of America; 3 Department of Rehabilitation Sciences, University of Texas Medical Branch, Galveston, Texas, United States of America; 4 Department of PMCH Epidemiology and Biostatistics, University of Texas Medical Branch, Galveston, Texas, United States of America; 5 Department of Internal Medicine, Sealy Center of Aging, University of Texas Medical Branch, Galveston, Texas, United States of America; Queen Elizabeth Hospital, Hong Kong

## Abstract

**Background:**

Little is known about the effect of provider continuity prior to the diagnosis of advanced lung cancer and end-of-life care.

**Methods:**

Retrospective analysis of 69,247 Medicare beneficiaries aged 67 years or older diagnosed with Stage IIIB or IV lung cancer between January 1, 1993 and December 31, 2005 who died within two years of diagnosis. We examined visit patterns to a primary care physician (PCP) and/or any provider one year prior to the diagnosis of advanced lung cancer as measures of continuity of care. Outcome measures were hospitalization, ICU use and chemotherapy use during the last month of life, and hospice use during the last week of life.

**Results:**

Seeing a PCP or any provider in the year prior to the diagnosis of advanced lung cancer increased the likelihood of hospitalization, ICU care, chemotherapy and hospice use during the end of life. Patients with 1–3, 4–7 or >7 visits to their PCP in the year prior to the diagnosis of lung cancer had 1.0 (reference), 1.08 (95% CI; 1.04–1.13), and 1.14 (95% CI; 1.08–1.19) odds of hospitalization during the last month of life, respectively. Odds of hospice use during the last week of life were higher in patients with visits to multiple PCPs (OR 1.10: 95% CI; 1.06–1.15) compared to those whose visits were all to the same PCP.

**Conclusion:**

Provider continuity in the year prior to the diagnosis of advanced lung cancer was not associated with lower use of aggressive care during end of life. Our study did not have information on patient preferences and result should be interpreted accordingly.

## Introduction

Outpatient provider continuity is central to the “medical home” concept of the *Patient Protection and Affordable Care Act* and is key to good medical care[1]. It is associated with improved patient satisfaction,[2] increased use of appropriate preventive health services[3]–[7], greater medication compliance, lower hospitalization rates,[8]–[12] less emergency department use[13] and fewer duplicate tests.[14] Moreover, continuity of care with a primary care physician (PCP) has shown substantial reductions in mortality among older adults.[15]

Continuity is beneficial for cancer patients both prior to and after diagnosis. Pre-diagnosis continuity leads to diagnoses at earlier stages.[16] PCP continuity after cancer diagnosis increases the likelihood of receiving guideline concordant therapy,[17] decreases the likelihood of emergency room visits in the last six months of life,[18] and increases the likelihood of dying at home.[19] Patients visited by their PCP during their last hospitalization (outpatient to inpatient provider continuity) are less likely to receive Intensive Care Unit (ICU) care.[20]

Studies of the effects of continuity on end-of-life care in cancer patients are primarily limited to patients already diagnosed with cancer. We were interested in whether the continuity established prior to this life- and care-altering event (diagnosis of advanced lung cancer) has enduring effects on the end-of-life medical care these patients received. Our hypothesis was that provider continuity prior to the diagnosis of advanced cancer is associated with lower hospitalization, less ICU use, less chemotherapy use and higher hospice use at the end of life. These measures are considered indicators of potentially appropriate care. We used a national sample of newly-diagnosed advanced lung cancer patients to examine the effect of provider continuity prior to the diagnosis of advanced lung cancer on end-of-life medical care.

## Methods

### Data Source

This is a retrospective study of advanced lung cancer patients identified from the linked Surveillance, Epidemiology and End Results (SEER)-Medicare database for the years 1993–2005. We included the 13 SEER registry geographic regions: the states of Connecticut, Hawaii, Utah, New Mexico, Iowa, California, Kentucky, Louisiana and New Jersey; rural Georgia; and the municipalities of Detroit, MI; Seattle, WA; and Atlanta, GA. For all incident cancers diagnosed in these areas, the SEER registries collect information on patient demographics, tumor characteristics, stage at diagnosis, date of diagnosis, therapy received within four months of diagnosis, and date and cause of death.

Through a collaborative project between the National Cancer Institute and the Centers for Medicare and Medicaid Services (CMS), entitlement information and claims data from the Medicare program were linked to the SEER data for cancer patients aged 65 and older. Medicare eligibility could be identified for 93% of SEER patients aged 65 and older.

Data from multiple files were used for this study: 1) the Patient Entitlement and Diagnosis File (SEER registry data and Medicare entitlement information); 2) Medicare Provider Analysis and Review file (MEDPAR, hospital inpatient and skilled nursing facility stays); 3) Outpatient Standard Analytic File (hospital outpatient services); 4) Physician/Supplier File (physician and other medical services); and 5) Hospice File.

### Study Cohort

Eligible subjects selected from the Patient Entitlement and Diagnosis File included patients who were: 1) diagnosed with stage III B or stage IV lung cancer from 1993 – 2005 (N = 143,515), 2) 67 years or older at the time of diagnosis (N = 113,183), 3) enrolled in Medicare Parts A and B for at least two years prior to death (N = 76,434), and 4) died within two years of diagnosis and had continuous enrollment in Parts A and B until death (N = 69,427). To have completeness of information in the Medicare files of these patients, we excluded those enrolled in an HMO at any time from two years before date of diagnosis through date of death.

### Measures

#### Patient characteristics

Patients' sociodemographic characteristics were obtained from the SEER data and included the following variables: age (67–74, 75–84, ≥85 years), gender (male, female), race/ethnicity (non-Hispanic white, Black, Hispanic, Other), diagnosis year and SEER geographic region. Socioeconomic status was based on whether the patient was eligible for state buy-in coverage provided by the Medicaid program for at least one month during the year of diagnosis. Comorbidity was measured with the Charlson comorbidity score (0, 1, 2, ≥3) using all Medicare claims from the year prior to diagnosis.[9] Survival time after diagnosis was measured in months (<1, 2–3, 4–6, 7–12, 13–18, 19–24). We also included the number of hospitalizations (0, 1, ≥2) in the 13–24 months before diagnosis as an independent variable.

#### Definition of study variables

HCFA Common Procedure Terminology (CPT) evaluation and management codes 99201 to 99205 (new patient) and 99221 to 99215 (established patient encounters) were used to establish outpatient visits. Individual providers were determined using the Unique Provider Identification Number (UPIN). We examined visits to PCPs and to any provider. PCPs included those in: family medicine, general practice, internal medicine and geriatrics. For female patients, OB/Gyn was included. Any provider was defined as a primary physician or an internal medicine specialist. To avoid overestimation of visits and continuity, we excluded visits that occurred one month prior to the diagnosis of lung cancer. Visits between months −2 to −13 constitute a year prior to diagnosis (herein referred to as the year prior to diagnosis). In some analyses we assessed visits in months −13 to −24 prior to diagnosis.

#### Provider continuity

We examined visit patterns for PCPs and for any provider separately as a measure of continuity. Visits to a PCP were defined as the total number of PCP visits in the year prior to diagnosis. (0, 1–3, 4–7, >7). Number of PCPs was defined as the number of unique PCPs seen in the year prior to diagnosis. Having more than one PCP was defined as seeing multiple PCPs.

Visits to any provider was defined as the total number of visits to any physician in the year prior to diagnosis (0, 1–3, 4–7, >7). Number of any providers seen was defined as the number of unique providers seen in the year prior to diagnosis (unique UPINs). Seeing more than one physician was defined as having multiple providers. We assigned a PCP or a provider to a given patient with whom he/she had the majority of outpatient visits in the year prior to diagnosis of advanced lung cancer.

We also used the time exposure method of continuity as defined by Wolinsky et al.[21] To calculate this measure, we expanded the pre-diagnosis period to two years and defined continuity (yes/no) as having visited the same physician twice within any eight months during that time.

#### Outcomes

Our outcome of interest was medical care during the end of life. We examined hospitalization, ICU use, and chemotherapy use during the last month of life, and hospice use during the last week of life. The first three were considered indicators of potentially aggressive (overtreatment) end of life care and hospice use as appropriate end of life care.

The study was approved by the Institutional Review Board of the University of Texas Medical Branch, Galveston, TX. De-identified data provided by SEER-Medicare were analyzed and no prior informed consent was required. The manuscript was approved by SEER-Medicare for anonymity prior to submission for publication.

#### Statistical Analysis

The chi square test was used to compare end-of-life care by patient characteristics. Multivariate logistic regression analyses were used to assess whether different continuity measures affected end-of-life care (yes/no). Both patient characteristics (including age, gender, race, diagnosis year, geographic region, comorbidity, survival time, number of hospitalizations in the 13–24 months before diagnosis) and continuity measures (having a PCP, number of PCPs, total number of visits to PCP, total number of visits to assigned PCP, time exposure method of continuity) were added to the final model. All analyses were performed with SAS version 9.2 (SAS, Inc., Cary, NC). All reported p-values were two-sided and p<0.05 was considered statistically significant.

## Results

Table 1 presents the baseline characteristics of the cohort and medical care measures (hospitalization during the last month of life, ICU use during the last month of life, chemotherapy use during the last month of life, and hospice use during the last week of life) in 69,427 patients diagnosed with advanced lung cancer who were followed for 24 months. Younger patients, males, non-whites and those with low socioeconomic status were more likely to be hospitalized and to receive ICU care during the last month of life. Hospice use was more common in females, non-Hispanic whites and those with higher socioeconomic status. Patients with lower comorbidity scores were less likely to be hospitalized or to receive ICU care.

**Table 1:** Baseline characteristics and percent of patients experiencing end of life care measures in Medicare patients with advanced lung cancer, 1993–2005.

		End of life care measures,% yes			
Characteristic^3^	N	Hospitalized^1^	ICU^1^	Chemotherapy^1^	Hospice^2^
All Subjects	69427	50.4	13.0	12.8	45.6
Age at Diagnosis (yrs)					
66–74	30169	53.5	14.5	16.6	44.5
75–84	31478	49.2	12.5	11.3	46.6
> = 85	7780	43.1	9.4	3.9	46.0
Gender					
Male	37718	51.9	13.8	14.0	42.7
Female	31709	48.5	12.0	11.3	49.1
Race					
Non-Hispanic White	58453	49.6	12.3	13.3	47.1
Black	2227	54.5	16.3	9.0	39.0
Hispanic	5854	52.7	17.2	12.2	42.4
Other	2893	56.3	18.1	10.4	31.6
Low Socioeconomic Status					
No	58638	50.1	12.4	13.6	47.1
Yes	10789	52.1	16.2	8.2	37.8
Charlson Comorbidity Score					
0	35795	48.6	11.6	13.3	46.9
1	18663	51.6	13.6	13.2	46.3
2	8237	51.9	15.3	11.8	43.2
≥3	6732	54.6	16.3	10.1	40.3
Times hospitalized in the 13–24 months before diagnosis					
0	57297	50.0	12.7	13.2	45.9
1	8054	51.2	13.8	11.2	45.2
>2	4076	53.8	16.1	10.3	42.2
Survival time (months)					
0–1	15445	45.2	13.1	4.3	26.1
2–3	19052	59.6	16.0	14.0	46.6
4–6	13373	48.8	11.7	16.6	51.9
7–12	13304	48.0	11.0	15.9	54.3
13–18	5755	46.2	11.3	15.4	55.8
19–24	2498	43.1	11.0	13.4	56.2
SEER site					
Connecticut	5916	50.3	9.5	12.9	38.5
Detroit	8308	57.4	16.0	12.1	51.0
Hawaii	1149	49.9	11.9	8.9	38.1
Iowa	6604	45.6	6.2	11.0	52.0
New Mexico	1594	43.0	11.7	10.2	50.8
Seattle	5201	44.9	9.0	12.8	41.0
Utah	1259	36.1	9.2	9.3	48.8
Atlanta	2562	48.8	10.5	16.2	47.1
Rural Georgia	232	50.4	9.9	16.8	44.8
Kentucky	5248	49.3	13.4	13.2	51.8
Louisiana	4035	51.9	10.9	11.7	50.6
New Jersey	7446	55.6	16.9	17.8	45.8
California	19873	50.2	15.8	12.1	41.5
Diagnosis Year					
1993	3394	51.4	8.9	7.3	28.3
1994	3447	47.7	9.5	8.8	33.9
1995	3472	50.0	9.7	8.5	33.8
1996	3440	49.8	10.1	10.1	37.5
1997	3311	50.8	11.4	11.3	41.4
1998	3397	47.9	10.5	11.9	43.6
1999	3257	49.3	11.6	13.1	45.7
2000	7219	50.7	12.4	14.2	46.5
2001	7675	50.8	14.0	14.9	47.0
2002	7739	49.9	14.0	14.8	48.9
2003	8023	51.4	15.4	14.5	49.9
2004	7794	50.4	14.9	14.3	52.1
2005	7259	51.7	15.9	12.2	54.1

1  =  use in last month of life.

2  =  use in last week of life. ICU, Intensive Care Unit; SEER, Surveillance, Epidemiology and End Results.

3  = All differences in the four measures of end of life care by characteristics were statistically significant with p<0.0001.

There was large variation in the receipt of aggressive end-of-life care and hospice care across SEER sites. Relative to other areas, hospitalization was more common in the Detroit metropolitan area; ICU use and chemotherapy use during the last month of life were more common in New Jersey; and hospice use was more common in Iowa. ICU use increased from 8.9% in 1993 to 15.9% in 2005 (p<0.0001). Similarly, hospice use increased from 28.3% in 1993 to 54.1% in 2005 (p<0.0001). Aggressive end-of-life care was more common in patients with shorter survival time. Patients who died within three months of diagnosis of advanced lung cancer were more likely to be hospitalized and receive ICU care during the last month of life. By contrast, hospice use was more common in those with longer survival time: 26.1% in those who survived < 1 month compared to 56.2% in those who survived > 18 months.

Table 2 presents the bivariate analyses for several measures of provider continuity and end-of-life care. Seeing a PCP in the year prior to a diagnosis of advanced lung cancer increased the likelihood of hospitalization, ICU care during the last month of life, as well as hospice use during the last week of life. The association between end-of-life care and number of PCPs seen and number of outpatient visits to PCPs was inconsistent.

**Table 2:** Provider continuity in the year prior to diagnosis of advanced lung cancer and end-of-life care.

		End of life care measures,% yes			
Continuity Measure	N	Hospitalized^1^	ICU^1^	Chemotherapy^1^	Hospice^2^
All Subjects	69427	50.4	13.0	12.8	45.6
Saw a PCP					
No	22106	47.3	12.3	11.1	41.4
Yes	47321	51.8	13.3	13.6	47.6
Number of PCPs seen					
0	22106	47.3	12.3	11.1	41.4
1	33289	51.6	13.2	13.7	46.9
>1	14032	52.4	13.7	13.3	49.3
Total number of visits to PCP					
0	22106	47.3	12.3	11.1	41.4
1–3	20193	50.0	12.4	14.0	48.2
4–7	17229	52.5	13.0	13.8	48.2
>7	9899	54.3	15.9	12.3	45.2
Number of visits to an assigned PCP (N = 47321)					
1–3	23328	50.3	12.5	14.0	48.6
4–7	16445	52.4	13.3	13.7	48.0
>7	7548	55.0	16.3	12.2	43.5
Time exposure method continuity of PCP^3^					
No	25745	47.8	12.2	11.5	42.2
Yes	43682	51.9	13.5	13.6	47.6
Saw any provider					
No	13300	43.3	10.8	9.2	39.7
Yes	56127	52.1	13.5	13.6	47.0
Number of any provider seen					
0	13300	43.3	10.8	9.2	39.7
1	21880	50.5	12.5	12.4	45.4
>1	34247	53.1	14.2	14.5	48.0
Total number of visits to any provider					
0	13300	43.3	10.8	9.2	39.7
1–3	17226	48.9	11.8	13.1	47.1
4–7	18728	51.7	12.6	13.7	48.4
>7	20173	55.0	15.9	14.0	45.7
Number of Visits to a assigned provider (N = 56127)					
1–3	25325	49.8	12.1	13.7	48.3
4–7	20875	53.0	13.7	13.9	47.6
>7	9927	55.9	16.7	12.8	42.7
Time exposure method continuity of any provider^3^					
No	16727	44.4	10.9	10.2	40.8
Yes	52700	52.3	13.7	13.6	47.1

1  =  in last month of life.

2  =  in last week of life.

ICU, intensive care unit; PCP, primary care physician.

3 = Time exposure method of continuity is defined as having visited the same physician twice within any eight months over the two years prior to the diagnosis of lung cancer

Table 3 shows the results of multivariable analyses of the association between provider continuity and end-of-life care measures. Seeing a PCP or any provider and having continuity with a PCP or any provider was associated with increased risk of hospitalization, ICU use and chemotherapy use during the last month of life, as well as hospice use during the last week of life. The odds of hospitalization and ICU use in the last month of life increased stepwise with an increase in number of visits. For example patients with 1–3, 4–7 or >7 visits to their PCP in the year prior to the diagnosis of lung cancer had 1.0 (reference), 1.08 (95% CI; 1.04–1.13), and 1.14 (95% CI; 1.08–1.19) odds of hospitalization during the last month of life, respectively. The results were similar for analyses limited to patients with an identifiable PCP. There was no clear association with hospice use during the last week of life, except for higher odds in patients with outpatient visits to multiple PCPs compared to those whose visits were all to the same PCP. Patients who made more visits (≥7) to their assigned PCP had 0.91 (0.86–0.96) lower odds of hospice use during the last week of life. There was no difference in end-of-life care measures using the time exposure method.

**Table 3:** Multivariable analyses of the associations between provider continuity measures and end-of-life care.

	End of life care measures			
	Hospitalized^1^ OR (95% CI)	ICU^1^ OR (95% CI)	Chemotherapy^1^ OR (95% CI)	Hospice^2^ OR (95% CI)
**PCP**				
Saw a PCP				
No	**0.82 (0.79,0.84)**	**0.88 (0.84,0.93)**	**0.77 (0.73,0.81)**	**0.84 (0.82,0.87)**
Yes	ref	ref	ref	ref
Number of PCPs seen				
0	**0.82 (0.80,0.85)**	**0.89 (0.84,0.94)**	**0.77 (0.72,0.81)**	**0.87 (0.84,0.90)**
1	ref	ref	ref	ref
> 1	1.04 (1.00,1.08)	1.03 (0.97,1.10)	1.00 (0.94,1.06)	**1.10 (1.06,1.15)**
Total visits to PCP				
0	**0.86 (0.83,0.89)**	**0.91 (0.85,0.96)**	**0.78 (0.73,0.82)**	**0.85 (0.81,0.88)**
1–3	ref	ref	ref	ref
4–7	**1.08 (1.04,1.13)**	0.99 (0.93,1.06)	1.03 (0.97,1.10)	1.02 (0.97,1.06)
>7	**1.14 (1.08,1.19)**	**1.15 (1.07,1.24)**	1.01 (0.94,1.10)	0.97 (0.92,1.02)
Number of visits to assigned PCP				
1–3	ref	ref	ref	ref
4–7	**1.07 (1.03,1.12)**	1.01 (0.95,1.07)	1.05 (0.99,1.11)	1.00 (0.96,1.05)
>7	**1.16 (1.09,1.22)**	**1.17 (1.08,1.26)**	1.02 (0.94,1.12)	**0.91 (0.86,0.96)**
Time exposure method continuity of PCP^3^				
No	0.92 (0.83,1.02)	0.93 (0.79,1.09)	1.09 (0.94,1.26)	0.94 (0.84,1.04)
Yes	ref	ref	ref	ref
**Any Provider**				
Saw any provider				
No	**0.68 (0.65,0.71)**	**0.77 (0.72,0.82)**	**0.61 (0.57,0.66)**	**0.8 (0.76,0.83)**
Yes	ref	ref	ref	ref
Number of any providers seen				
0	**0.72 (0.68,0.75)**	**0.81 (0.75,0.86)**	**0.67 (0.62,0.72)**	**0.83 (0.79,0.87)**
1	ref	ref	ref	ref
> 1	**1.1 (1.07,1.14)**	**1.09 (1.04,1.15)**	**1.18 (1.12,1.24)**	**1.09 (1.05,1.13)**
Total visits to any provider				
0	**0.75 (0.71,0.78)**	**0.83 (0.77,0.89)**	**0.67 (0.62,0.72)**	**0.81 (0.77,0.85)**
1–3	ref	ref	ref	ref
4–7	**1.12 (1.07,1.17)**	1.04 (0.98,1.11)	**1.10 (1.04,1.18)**	**1.06 (1.02,1.11)**
>7	**1.26 (1.21,1.32)**	**1.24 (1.16,1.32)**	**1.23 (1.15,1.32)**	1.01 (0.96,1.05)
Number of visits to assigned provider				
1–3	ref	ref	ref	ref
4–7	**1.13 (1.09,1.18)**	**1.08 (1.02,1.15)**	**1.1 (1.04,1.16)**	1 (0.96,1.04)
>7	**1.25 (1.19,1.31)**	**1.26 (1.17,1.35)**	**1.12 (1.04,1.2)**	**0.89 (0.84,0.93)**
Time exposure method continuity of any provider^3^				
No	0.92 (0.83,1.02)	0.88 (0.74,1.05)	0.97 (0.84,1.14)	**0.86 (0.77,0.96)**
Yes	ref	ref	ref	ref

ICU, intensive care unit; PCP, primary care physician; OR, odds ratio. CI, confidence interval. All models adjusted for age at diagnosis, gender, race, low socioeconomic status, SEER site, diagnosis year, survival time, Charlson comorbidity score and number of times hospitalized in the 13–24 months prior to diagnosis. Bolded values indicate p < 0.05.

3 = Time exposure method of continuity is defined as having visited the same physician twice within any eight months over the two years prior to the diagnosis of lung cancer

One potential confounder in analysis of pre-diagnosis continuity of care with post-diagnosis care is that earlier provider visits, precipitated by symptoms of the yet-undiagnosed cancer, may affect the estimation of true continuity of care. To address that possibility, we repeated all analyses using measures of provider visits generated in the period 13–24 months prior to the diagnosis of advanced lung cancer. Those analyses produced very similar results to those shown for the analysis using the 2–13 months prior to diagnosis.

## Discussion

Prior studies on continuity and end-of-life care in patients with cancer have focused on patient-provider patterns following cancer diagnosis.[18]–[20]; [22]–[24] Continuity of care after diagnosis seems to matter. However, whether the continuity established prior to diagnosis of advanced lung cancer has an effect on end-of-life medical care is unknown. We examined associations between several measures of provider continuity prior to the diagnosis and medical care received during end of life.

Provider continuity in the year prior to the diagnosis of advanced lung cancer was not substantially associated with less aggressive medical care during end-of-life care. However, survival time had a significant effect on end-of-life care. Patients with shorter survival were more likely to receive aggressive care whereas those with longer survival were likely to receive hospice care at the end of life. Long term survivors likely represent individuals with lung cancer sensitive to the standard initial chemotherapy and radiotherapy. These individuals are unlikely to benefit from additional treatment later in the course of their disease; thus, they are more likely to opt for hospice care. Moreover, longer survivors also had more time to cope with their terminal illness and perhaps are better prepared for end of life.

Hospice care focuses on palliative care and symptom management rather than disease treatment. Our study showed both aggressive and palliative care increased over time, similar to prior reports [20]; [25]. Patients are choosing both types of care, rather than one or the other, regardless of provider continuity. These results are similar to a Kronman et al. study which showed no clear effect of PCP visits on hospice care.[26] A recent randomized control trial showed that patients with advanced lung cancer who received early palliative care consultation in addition to standard treatment were less likely to receive aggressive care during end of life and lived 2.6 months longer than those who received standard treatment alone [27]. The benefits seen in this study likely arise from better understanding of their disease process and more time to declare their end of life choices.

Our findings are consistent with the study by Kronman et al. of decedent Medicare beneficiaries; compared to patients with no PCP visits during the 7–18 months prior to death, those with ≥9 PCP visits were hospitalized more frequently (0.9 versus 1.3 admissions), but they averaged 1.9 fewer days in the hospital during the last 6 months of life.[26] We did not examine the total number of days spent in the hospital, but whether patients had any hospital or ICU stay during the last month of life.

Inherent to any continuity of care measure, whether based on visit pattern or indices, is the assumption that higher continuity of care leads to greater patient-physician trust. End-of-life decisions are often based on value, preferences and trust between patient and provider. A recent mixed method study of cancer patients showed that “experiencing continuity” involves receiving consistent time and attention, knowing future expectations, managing family consequences, coping between service contacts and believing that nothing has been overlooked.[28] Higher “experienced continuity” is associated with lower care needs. These aspects of continuity are not captured in claims data.

Our study has several limitations. Information on patient preferences and appropriateness of clinical care is not available in claims data. These findings are limited to fee-for-service Medicare beneficiaries and are not generalizable to other populations. A patient's preferences change following diagnosis of a terminal illness and during the course of his or her disease. Moreover, a recently-diagnosed cancer patient tends to see several new providers in multiple care settings. Our study is limited to advanced lung cancer patients with generally poor survival and the results may not be applicable to patient diagnosed with other cancers. Lack of detailed information on patient's medical and social status in claims data may have added unmeasured confounding and potential explanation for lack of association between prior continuity and end of life care.

In conclusion, our study showed no consistent association between provider continuity prior to diagnosis of advanced lung cancer and end of life care. Continuity did not substantially reduce potentially inappropriate or increase potentially appropriate care during end of life.
